# Passive imaging through inhomogeneous scattering media

**DOI:** 10.1038/s41598-024-66449-4

**Published:** 2024-07-09

**Authors:** Yaoming Bian, Fei Wang, Haishan Liu, Haiming Yuan, Siteng Li, Wenxin Huang, Guohai Situ

**Affiliations:** 1 Shanghai Institute of Optics and Fine Mechanics, Chinese Academy of Sciences, Shanghai, 201800 China; 2 Center of Materials Science and Optoelectronics Engineering, University of Chinese Academy of Sciences, Beijing, 100049 China; 3 Hangzhou Institute for Advanced Study, University of Chinese Academy of Sciences, Hangzhou, 310024 China

**Keywords:** Imaging and sensing, Atmospheric optics

## Abstract

According to the atmospheric scattering model (ASM), the object signal’s attenuation diminishes exponentially as the imaging distance increases. This imposes limitations on ASM-based methods in situations where the scattering medium one wish to look through is inhomogeneous. Here, we extend ASM by taking into account the spatial variation of the medium density, and propose a two-step method for imaging through inhomogeneous scattering media. In the first step, the proposed method eliminates the direct current component of the scattered pattern by subscribing to the estimated global distribution (background). In the second step, it eliminates the randomized components of the scattered light by using threshold truncation, followed by the histogram equalization to further enhance the contrast. Outdoor experiments were carried out to demonstrate the proposed method.

## Introduction

Imaging through inhomogeneous and dense scattering media is widely encountered in the various fields of science and engineering. However, the inherent scattering properties of scattering media along the imaging pathway prohibit a clear image of the scene within or behind it to be formed^1^. On one hand, the scattering particles absorb or scatter light directly from the scene (i.e., signal light), leading to a reduction in signal intensity. On the other hand, these particles also scatter ambient light that does not carry any information of interest, resulting in strong interference noise superposing on the raw images captured by a camera.

Many methods have been proposed, attempting to address this challenging problem. In particular, recent advancement in gating technologies^2–6^, point-wise scanning strategies^7^, wavefront shaping^8^, optical transmission matrix measurement^9^, and speckle correlations^10–12^ has received a lot of attentions. But all these advanced techniques rely on the use of coherent light sources as active illumination. Thus, one can apply coding to the illumination, leveraging the efficiency of information extraction. In the cases where coherent active illumination is not possible, one has to mainly rely on defogging algorithms in addition to the optimization of the imaging system^13^. So far, algorithms depending on image depth priors^14^, dark channel priors^15^, histogram equalization^16^, Retinex-based^17^, wavelet transform^18^ and data-driven deep learning^19–22^ have been proposed to enhance the contrast of the captured raw images. These defogging algorithms more or less are developed on the base of the atmospheric scattering model (ASM). This model assumes that the attenuation of the object signal exponentially diminishes as the imaging distance increases^23,24^. This assumption does not hold when dealing with inhomogeneous scattering media.

Here we address the challenge of imaging through inhomogeneous and dense scattering media. Our approach is to first extend the conventional ASM by introducing a spatially variant attenuation coefficient, and then develop a two-step defogging algorithm in accordance with it for image recovery.

## Methods

**Figure 1:**
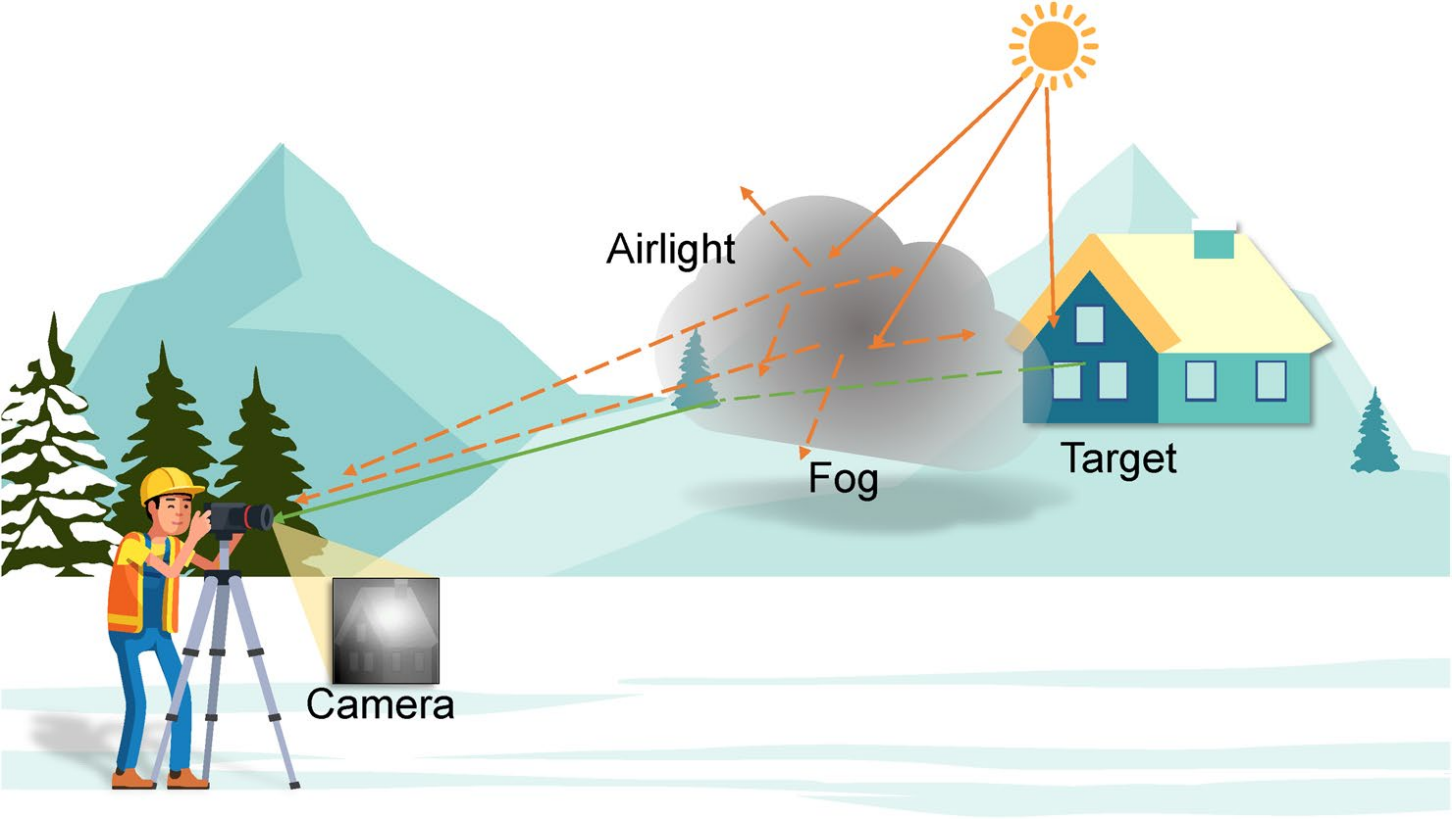
The schematic illustration of the scenario: imaging through inhomogeneous fog.

Now let us go into the technical details. The conventional ASM that describes the formation of a hazy image under sun light illumination can be expressed as^23,24^

1 $$\begin{aligned} I(x) = L_{\infty } \rho (x)\exp [-\alpha d(x)]+ L_{\infty } (1-\exp [-\alpha d(x)]), \end{aligned}$$

where *I*(*x*) is the hazy image, $L_{\infty }$ is the global atmospheric light, $\rho (x)$ is the albedo of a target scene, $\alpha$ is the attenuation coefficient of the atmosphere, and *d*(*x*) is the distance between the scene and the camera. The first term on the right-hand side of Eq. (1) represents the transmission attenuation of signal(green solid line in Fig. 1), and the second one is the superposition of airlight^24^(orange dashed line in Fig. 1), which is scattered by the fog and contains no target information. Equation (1) describes the phenomenon that the signal light attenuates exponentially as it propagates through the scattering media, whereas the airlight is the opposite. The scene is barely visible when the imaging distance *d* exceeds the range of visibility.

This equation can be rewritten as^24,25^

2 $$\begin{aligned} \begin{aligned} I(x) = J(x)t(x)+A[1-t(x)], \end{aligned} \end{aligned}$$

where $J(x)=L_\infty \rho (x)$ denotes the clear image without disturbance by scattering. $t(x)=\exp [-\alpha d(x)]$ is the transmission attenuation ratio, and *A* is the global airlight at an infinite distance, which is usually estimated from the sky area of a hazy image *I*(*x*). The purpose of defogging algorithms is to recover *J*(*x*) from *I*(*x*).

It is important to note that *t*(*x*) in Eq. (2) decays exponentially as a function of *d*(*x*) with a constant attenuation factor $\alpha$. $\alpha$ is independent of distance, indicating that the scattering medium is homogeneous. Nevertheless,this is not always the case with scattering media in real-world situations. As crudely illustrated in Fig. 1 , natural scattering media such as fog frequently look spatially inhomogeneous in terms of both thickness and density. The green solid line depicts the signal light from the target scene, and the dashed portion of it indicates the attenuation caused by the fog it propagates through. The orange dashed line is the airlight scattered by the fog, which does not provide any information about the target and can be regarded as additive noise. Taking into consideration the inhomogeneity of the fog, we can phenomenologically rewrite the ASM model as

3 $$\begin{aligned} I(x) = J(x)t'(x)+B(x)+R(x), \end{aligned}$$

where the transmission attenuation ratio is given by

4 $$\begin{aligned} t'(x)=\exp [-\alpha (x)d(x)]. \end{aligned}$$

Clearly, the attenuation coefficient $\alpha (x)$ now becomes a function of the spatial coordinates *x*. The second term in Eq. (3) is related to $A[1-t(x)]$ in Eq. (2), i.e., $B(x)=A[1-\bar{t}'(x)]$, where $\bar{t}'=\exp [-\bar{\alpha }d(x)]$ is the time averaged transmission attenuation ratio over the course of exposure, and $R(x)=A[1-\exp [-(\alpha (x)-\bar{\alpha })]d(x)]$ is the deviation from *B*(*x*) that accounts for the inhomogeneity of the scattering medium.

**Figure 2:**
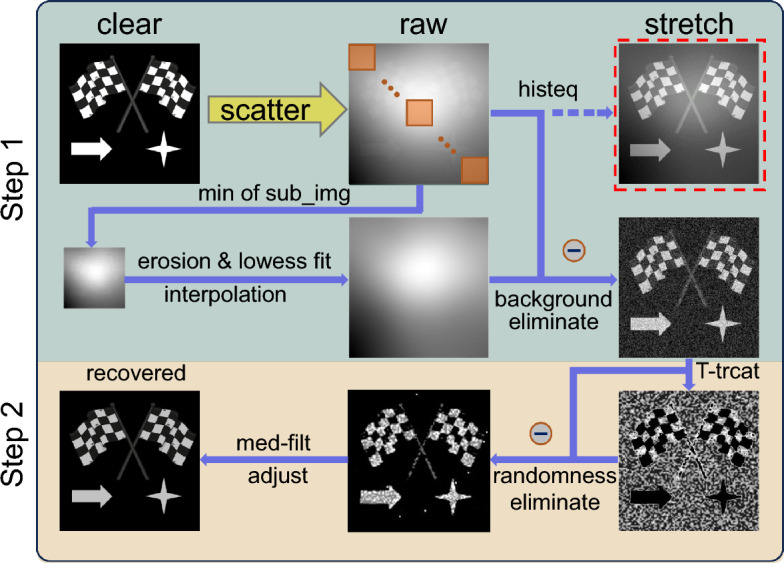
Pipeline of the proposed two-step defogging method. Step 1: The estimation of *B*(*x*). Step 2: The estimation of *R*(*x*) and subsequent image enhancement.

When an image captured by a camera, signal and noise are aliased. Therefore instead of estimating the attenuation coefficient $\alpha (x)$ directly, we estimated the magnitude of the additive noise (i.e., *B*(*x*) and *R*(*x*)) caused by it through a proposed two-step method. Our purpose then is to estimate and eliminate the noise *B*(*x*) and *R*(*x*) in Eq. (3), respectively. Based on the two-step method, in the first step, we estimate the statistical distribution of *B*(*x*) from the acquired hazy image *I*(*x*). The reason for this is the term *B*(*x*) is the most significant among the three when the scattering medium is optically thick. Here we adopt the space domain estimation inspired by Satat *et al.*’s time-of-flight-based time domain estimation method^26^.

As schematically illustrated by Step 1 in Fig. 2, the proposed method first divides the acquired hazy image into an array of $q\times q$ non-overlapping and connected sub-images, $I_i(x)$, of appropriate size, within which the scattering medium can be treated as homogeneous. This is reasonable in particular to take the mean of each sub-image, i.e., $b_i=\overline{I_i(x)}$, for the patches that only contain scattered light $B(x)+R(x)$. For the patches that contain the object signal $J(x)t'(x)$, which could be very weak though, we have demonstrated that estimating the noise level as the minimum value of the patch, i.e., $b_i=\textrm{min}I_i(x)$, is helpful for subsequent contrast enhancement^13^. This operation can be applied to all the other patches without the object signal $J(x)t'(x)$ because, in the case of our interest, it is the term *B*(*x*) that is the most significant as mentioned above. This means that the difference between $\textrm{min}I_i(x)$ and $\overline{I_i(x)}$ is insignificant. Thus we can take the minimum value $b_i=\textrm{min}I_i(x)$ of each sub-image as the estimation of the local averaged noise level $B_i(x)$, resulting in a noise map of $q\times q$ in size. Next we erode the estimated noise map in order to reduce the contribution of $J(x)t'(x)$, and then fit the spatial statistical distribution of noise map based on the assumption of macroscopic smooth variation of the scattering media^27^. Finally, we resize the downsampled noise map back to the original size by using, for instance, Bicubic interpolation, and subtracts this estimated noise $\hat{B}(x)$ from the hazy image *I*(*x*). In this way, the averaged noise *B*(*x*) in Eq. (3) can be dramatically reduced.

In the step 2 of the proposed algorithm we will eliminate the residual noise *R*(*x*) as shown in the orange patches in Fig. 2. Note that with the removal of the averaged noise *B*(*x*), the term $J(x)t'(x)$ in Eq. (3) should become significant, whereas the other term *R*(*x*) appears as small random disturbance superposing the signal term. Since we take the minimum value instead of the mean of each patch to estimate *B* in step 1, *R*(*x*) is positive. Thus this disturbance can be eliminated by pixel-wise subtracting it from $I_{\text{re}}$. To estimate *R*(*x*), we first define a threshold $\gamma = \mu + w \sigma$, where *w* is an adjustable weighting factor, and $\mu$ and $\sigma$ are the mean and standard deviation of $I_{\text{re}}$, respectively, and set the pixel of $I_{\text{re}}$ whose values are greater than $\gamma$ to be 0. And then we obtain an estimation, $\hat{R}(x)$, of *R*(*x*) by adjusting the threshold truncation through *w*, so that $\hat{I} = I_{\text{re}} - \hat{R}$. In this phase, the image of the target scene should be recovered. But we can further smooth it by standard median filtering, followed by histogram equalization to enhance the contrast owing to the transmission attenuation $t'(x)$. For more detailed information, one can see Algorithm 1.

**Algorithm 1:**
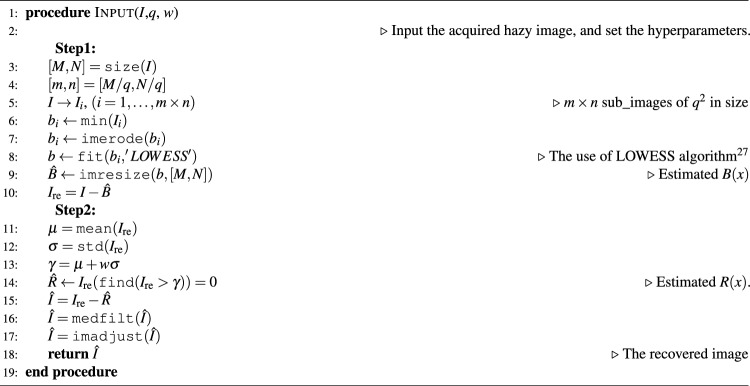
The proposed two-step algorithm.

## Experiment and results

We conducted outfield experiments to demonstrate the performance of the proposed method in natural environment. The scattering medium in this study was fog present in the imaging pathway. Fog existing in nature is inhomogeneous in terms of both thickness and density, and usually changes with time.

**Figure 3:**
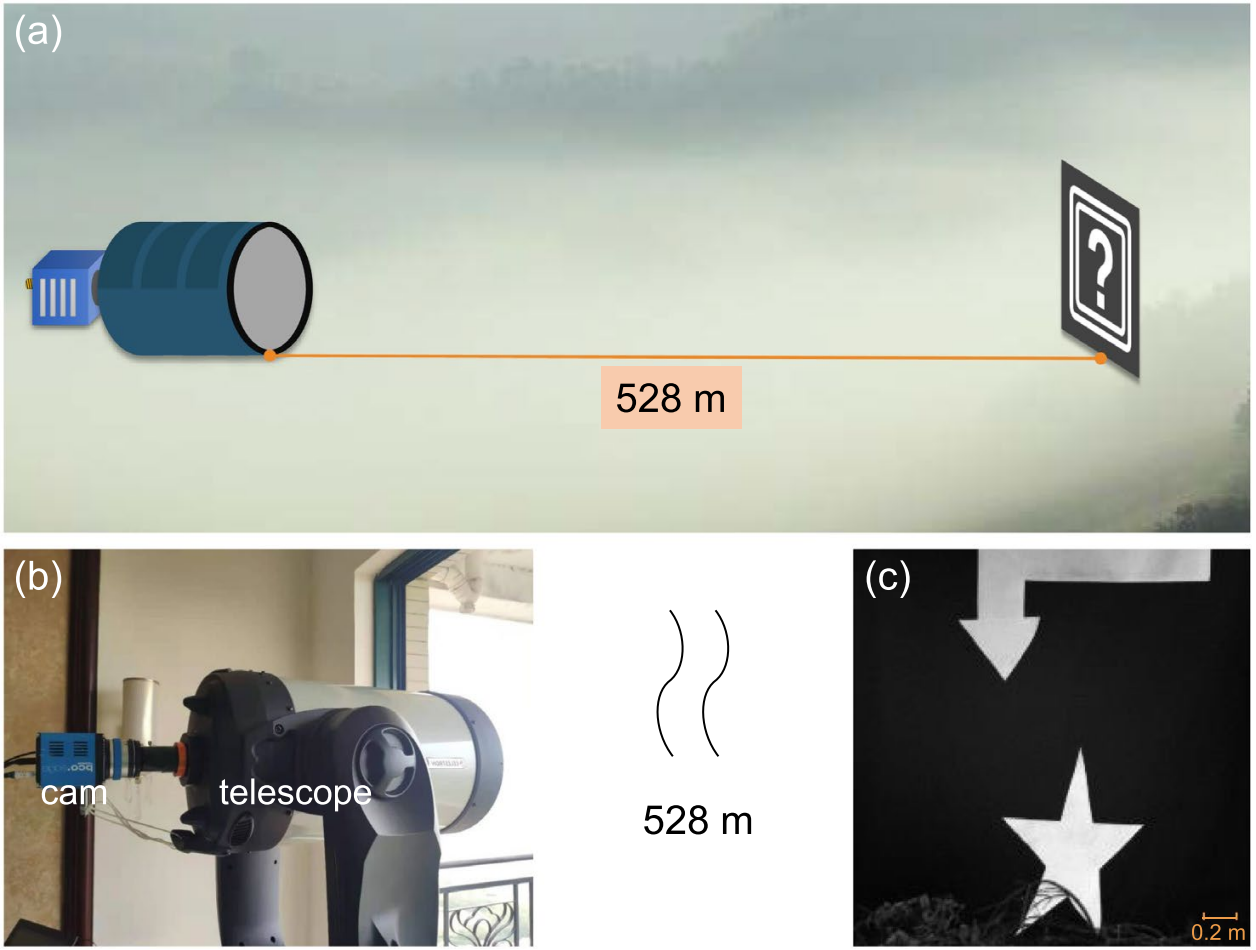
Outfield experiment. (**a**) Schematic illustration of the imaging scenario, (**b**) The imaging system, and (**c**) the image of the object captured by our imaging system in good weather. The object is a homemade black cloth with two white patterns attached on it and hanged on a cliff of 528 m away from the imaging system.

Figure 3a schematically depicts the scenario of our outfield experiments. As highlighted in this figure, the geometric imaging distance is 528 m. The imaging system (Fig. 3b) was a reflective telescope (CPC1100HD, Celestron) equipped with an sCMOS camera (Edge 4.2,PCO). The object to be imaged was a homemade black cloth with two white patterns attached on it. Its image taken in good weather is shown in Fig. 3c, and we use it as the ground truth in our analysis.

**Figure 4:**
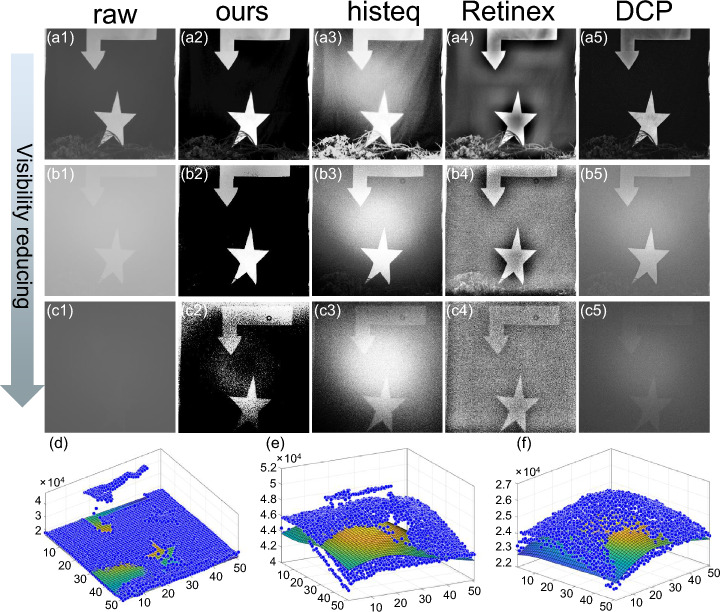
Experimental results. (**a1**–**c1**), the captured raw hazy images at ranges of different visibility. The images restored by using the proposed technique (**a2**–**c2**) histogram equalization (**a3**–**c3**), Retinex (**a4**–**c4**) and DCP (**a5**–**c5**). And (**d**–**f**), the estimated background noise of raw hazy images (**a1**–**c1**), where the dotted map in blue represent the raw hazy images.

**Figure 5:**
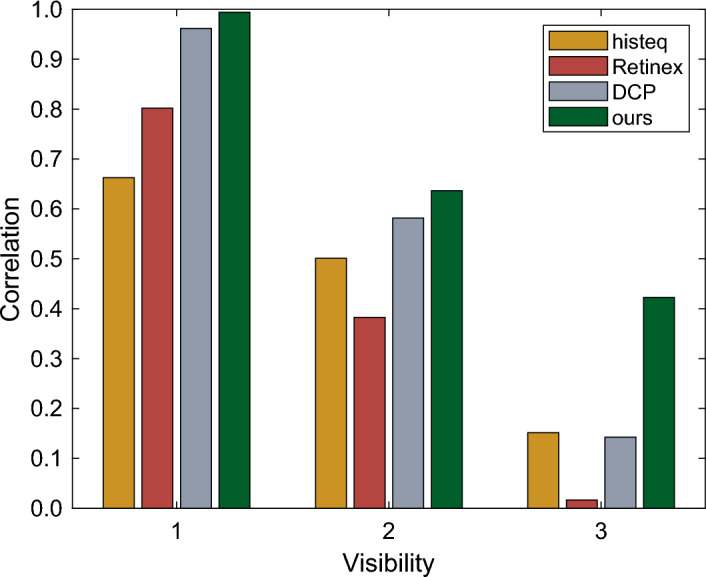
The Pearson correlation coefficients between the ground truth and the images restored by the four algorithms for comparison. Three groups of four values are associated with the three different ranges of visibility.

We captured hazy images of of the object in a cloud of fog with various ranges of visibility which is expressed as a function of the attenuation coefficient $\alpha (x)$ as $V= 2.996/\alpha$^13^. Then we restored the object images from these hazy ones. The main results are shown in Fig. 4. One can clearly see that the contrast of the raw hazy images is gradually decreased as the range of visibility reduces (Fig. 4a1–c1). In particular, one can see from Fig. 4b1 that the local contrast around the arrow and that around the star is different owing to the inhomogeneity of the fog. In the most serious case, the raw hazy image looks visibly uniformed as shown in Fig. 4c1 because the cloud of fog is so thick that the strong scattered light might undergo random walk. The images restored by using the proposed method are shown in Fig. 4a2–c2, with the corresponding estimated background scattering noise *B*(*x*) shown in Fig. 4d–f, respectively. One can see that even in the most serious case in our experiments the object image is revealed very well. This suggests that the ballistic light, although extincting exponentially with respect to $\alpha$, can still be detected and occupies the lower bits in the camera pixel values. But to the best of our knowledge, no existing algorithms can de-scatter such a strong background scattering noise. This can be clearly seen from the images recovered by some of the benchmark algorithms such as histogram equalization^28^ (Fig. 4a3–c3), Retinex^17^ (Fig. 4a4–c4), and the one based on dark channel prior (DCP)^15^ (Fig. 4a5–c5). The experimental results suggest that the proposed method outperforms the other three, effectively balancing high contrast and the preservation of the object details. In contrast, the object images revealed by the other three defogging algorithms have strong artifacts or inhomogeneous scattering noise.

To quantitatively evaluate the error, we calculate the Pearson’s correlation coefficient *r* between the ground truth (GT) and images restored by all the four algorithms. The results are plotted in Fig. 5. It clearly demonstrates that the image restored by our method correlates the best among the four with the ground truth, associated with the largest *r* value in all the cases.

## Conclusions

In summary, we have extended the conventional ASM to accommodate passive imaging through inhomogeneous and dense scattering media. This is done by recognizing that the scattered light can be written as summation of a averaged term and a fluctuation term. In accordance with this, we have developed a two-step method to estimate and eliminate these two terms sequentially.

Outfield experiments of passively imaging through inhomogeneous fog have been conducted to demonstrate the proposed method. The experimental results show that clear images can be revealed from the captured hazy ones, outperforming the commonly used methods such as histogram equalization, Retinex, and DCP. Although demonstrated it with the reconstruction of a binary image, the proposed method could be in principle adapted for the imaging of gray-scale objects through dense inhomogeneous scattering media. This will be investigated in a future study.

## Data Availability

Data underlying the results presented in this paper are not publicly available at this time but may be obtained from the authors upon reasonable request. Please contact by the e-mail: ymbian@siom.ac.cn. Link to data request: Data request link.
